# Cytokine Dynamics in Autism: Analysis of BMAC Therapy Outcomes

**DOI:** 10.3390/ijms242015080

**Published:** 2023-10-11

**Authors:** Dusan M. Maric, Danilo Vojvodic, Dusica L. Maric, Gordana Velikic, Mihajlo Radomir, Ivana Sokolovac, Debora Stefik, Nemanja Ivkovic, Sonja Susnjevic, Miljan Puletic, Oliver Dulic, Dzihan Abazovic

**Affiliations:** 1 Department for Research and Development, Clinic Orto MD-Parks Dr Dragi Hospital, 21000 Novi Sad, Serbia; ducamaric@gmail.com (D.M.M.); miki1991gr@gmail.com (M.R.); 2 Faculty of Stomatology Pancevo, University Business Academy, 26101 Pancevo, Serbia; miljenko.puletic@gmail.com; 3 Institute for Medical Research, Military Medical Academy, 11000 Belgrade, Serbia; vojvodic.danilo@gmail.com (D.V.); deborastefik@gmail.com (D.S.); 4 Medical Faculty of Military Medical Academy, University of Defense, 11000 Belgrade, Serbia; 5 Department of Anatomy, Faculty of Medicine, University of Novi Sad, 21000 Novi Sad, Serbia; ivkovicnemanja@yahoo.com; 6 Hajim School of Engineering, University of Rochester, Rochester, NY 14627, USA; 7 Center Sokolovac, 21000 Novi Sad, Serbia; i.sokolovac@gmail.com; 8 Department of Social Medicine and Health Statistics with Informatics, Faculty of Medicine, University of Novi Sad, 21000 Novi Sad, Serbia; sonja.susnjevic@mf.uns.ac.rs; 9 Department of Surgery, Faculty of Medicine, University of Novi Sad, 21000 Novi Sad, Serbia; oliver.dulic@mf.uns.ac.rs; 10 Biocell Hospital, 11000 Belgrade, Serbia; adzihan@gmail.com

**Keywords:** autism, autologous bone marrow aspirate concentrate (BMAC), cytokines, Th1, Th2

## Abstract

Autism spectrum disorder (ASD) has recently been linked to neuroinflammation and an aberrant immune response within the central nervous system. The intricate relationship between immune response and ASD remains elusive, with a gap in understanding the connection between specific immune mechanisms and neural manifestations in autism. In this study, we employed a comprehensive statistical approach, fusing both overarching and granular methods to examine the concentration of 16 cytokines in the cerebrospinal fluid (CSF) across each autologous bone marrow aspirate concentrate (BMAC) intrathecal administration in 63 male and 17 female autism patients. Following a six-month period post the third administration, patients were stratified into three categories based on clinical improvement: Group 1- no/mild (28 subjects), Group 2—moderate (16 subjects), and Group 3—major improvement (15 subjects). Our integrated analysis revealed pronounced disparities in CSF cytokine patterns and clinical outcomes in autism subjects pre- and post-BMAC transplantation. Crucially, our results suggest that these cytokine profiles hold promise as predictive markers, pinpointing ASD individuals who might not exhibit notable clinical amelioration post-BMAC therapy.

## 1. Introduction

Autism spectrum disorder (ASD) embody a set of neurodevelopmental disorders with multifactorial etiologies, where genetic, environmental, and immunological factors interplay in the disease’s manifestation [1]. While genetic mutations have been identified as significant contributors to ASD pathogenesis [2,3,4], the structural and functional deficiencies they induce in dendrites have cascading effects, affecting synaptic interactions and the broader neural network [5].

The role of immune dysregulation in ASD is becoming increasingly evident [6,7]. The gut–brain axis, acting bidirectionally, suggests that pathogenic microbiota and their metabolites can modulate brain function, with cytokines playing a pivotal role [8,9,10,11]. Such revelations have propelled the inclusion of immune parameters, like proinflammatory cytokines, in standard ASD diagnostics. 

Numerous studies have spotlighted the correlation between ASD and immune biomarkers, such as cytokines [12,13,14,15,16], immunoglobulins, and various antibodies [17,18,19,20,21,22,23,24,25,26]. Emerging research indicates that children with autism often exhibit elevated levels of proinflammatory cytokines and diminished levels of anti-inflammatory cytokines [12]. Despite this progress, a comprehensive understanding of the immunopathology and intricate cytokine networks in ASD remains elusive. Existing literature has been constrained by limited sample sizes, heterogeneous study designs, and varying stem cell administration protocols. 

While a few clinical trials and case studies have explored the effects of intrathecal bone marrow aspirate concentrate (BMAC) administration in ASD patients and demonstrated encouraging improvements in symptoms and severity of ASD, our study is the first to examine correlations between patients’ cerebrospinal fluid (CSF) cytokine profiles before and after BMAC therapy and their subsequent therapeutic outcomes [27,28,29,30,31]. We suppose that BMAC’s therapeutic effects may be attributed to the modulation of cytokine networks, which could make cytokine levels valuable biomarkers. This approach is innovative because it aims to elucidate the mechanisms underlying BMAC’s efficacy in ASD.

BMAC contains mesenchymal stem cells, hematopoietic stem cells, and growth factors extracted from a person’s bone marrow and associated with various biological disturbances, including chronic inflammation, oxidative stress, mitochondrial dysfunction, gastrointestinal issues, and immune dysregulation, also associated with some of the core ASD pathophysiologies. BMAC therapy harnesses the immunomodulatory and anti-inflammatory properties of BMAC to target the biological disturbances implicated in ASD pathogenesis [32]. 

Recognizing these gaps, our study aims to shed light on the effects of autologous BMAC transplantation on the cytokine profile of CSF in ASD patients. By leveraging advanced statistical methodologies, we aspire to unearth potential correlations between pre-treatment cytokine levels and therapeutic outcomes. Our hypothesis posits that improvements observed post-BMAC therapy might be attributed to modulated cytokine networks. If substantiated, this could herald a new era in ASD therapeutics, with cytokine levels in CSF serving as invaluable biomarkers for therapeutic monitoring, treatment prediction, and gauging the necessity for multiple treatments. 

## 2. Results

Samples were obtained from a total of 80 subjects, comprising 63 males (79%) and 17 females (21%), all aged between 2 and 17 years. All participants self-identified as Caucasians. Utilizing the Gilliam Autism Rating Scale (GARS) 3, we identified 87.5% of the participants as high-risk, with 85% at level 2 and 2.5% at level 3. The remaining 12.5% were categorized as low risk. A comprehensive breakdown of age, gender distribution, and detailed GARS-3 score functional levels can be found in Table 1. 

### 2.1. Classification

Patients received three intrathecal injections, each 30 days apart. The injection was administered immediately after the bone marrow processing. Over a six-month follow-up period, 59 patients were categorized based on their response to therapy as follows:

Group 1: 28 patients (47%) with no or mild improvement in ASD symptoms.

Group 2: 16 patients (27%) demonstrating moderate improvement in ASD symptoms.

Group 3: 15 patients (26%) showing major improvement in ASD symptoms.

There were no complications observed during or post-procedure. Some patients experienced adverse events during the procedure, which included mild headaches (7%), transient fever (3%), pain at the injection site (35%), and vomiting (5%). All these symptoms resolved within two hours.

### 2.2. Cytokine Levels

Cytokine levels were measured from the CSF collected prior to each BMAC transplantation. The initial measurement, taken before the first transplantation during the first procedure, serves as the baseline. Subsequent measurements occurred 30- and 60-days post-baseline, immediately preceding the second and third transplantations, respectively.

### 2.3. Cytokine Level Analyses

In our endeavor to understand the effects of BMAC transplantation on cytokines in ASD, we opted for comprehensive analyses stemming from two analytical frameworks. 

#### 2.3.1. Good Clinical Response Post Autologous BMAC Administration Associated with a Decreased Average CSF Concentration of Inflammatory and Th1 Cytokines

In baseline samples (1st procedure or infusion) obtained prior to the initial BMAC transplantation, a salient observation was the lower IL27 concentration in the no/mild improvement group (Group 1, Table 2). Intriguingly, average baseline concentrations of TNFα, IL2, IL6, IL9, IL12, IL13, IL17, IL1β, and IFNγ were virtually indistinguishable between Groups 1 and 3. 

During the second procedure, Group 3 displayed a unique cytokine concentration profile. Notably, this group exhibited reduced levels of IL1β, TNFα, IL2, IL12, and IFNγ. Furthermore, compared to Group 1, Group 3 had diminished levels of IL13 and IL17, while IL21 was notably decreased when juxtaposed with Group 2 (Table 2 and Table 3). Additionally, serial sample analysis after the inaugural transplantation showed a marked elevation in Th1, IL10, IL13, IL9, and IL17 exclusively in Group 1 (Table 4). Such variations in cytokine concentrations were not observed in Groups 2 and 3. 

By the time of the third procedure, cytokine levels among the groups converged, closely mirroring the baseline readings (Table 2). Any minor variations in cytokine concentrations between the initial and final procedures might be attributed to the dysregulation of modulatory pathways, as opposed to a systemic inflammatory response. 

#### 2.3.2. Analysis of Cytokine Concentration Trends across Procedures

We conducted an in-depth analysis, comparing cytokine levels between groups for each administration round as outlined in Table 5. Additionally, we analysed the differences between procedures within individual patient groups, as detailed in Table 6, utilizing the Kruskal–Wallis test. Our investigation uncovered pronounced disparities in cytokine concentrations before the second BMAC transplantation. Specifically, between Groups 1 and 3, distinctions were noted for GM-CSF, TNFα, IL1β, IL2, IFNγ, IL10, IL13, IL17, IL9, and IL21. Meanwhile, differences between Groups 2 and 3 were discernible for IL2 and IL10 (Table 5). Further analysis of the 2nd versus 1st administration procedures highlighted that only Group 1 manifested significant increases in GM-CSF, IL2, IL12, IFNγ, IL17, and IL27 (Table 2 and Table 6). However, when comparing cytokine concentrations from the 3rd to the 2nd or the 3rd to the 1st administration procedures, no notable statistical differences emerged among groups with varying clinical responses.

These observations reinforce the hypothesis that dysregulation of modulatory pathways transpired, rather than systemic inflammatory responses.

#### 2.3.3. Direction of Cytokine Changes across Administration Intervals

Table 7 presents the direction of cytokine changes for distinct classification groups at varied administration intervals. Specifically, changes during the 2nd versus the 1st, the 3rd versus the 2nd, and the 3rd versus the 1st administration. We used the Wilcoxon rank test to detect how the levels of one cytokine might move between the treatments. Significance levels are as follows for a *p* < 0.05, it is flagged with one star (*), for a *p*-value less than 0.01, it is flagged with 2 stars (**), for a *p*-value less than 0.001, it is flagged with three stars (***), and if a *p* < 0.0001, it is flagged with four stars (****). Not significant values are marked as “ns”.

For the 2nd versus 1st administration comparison, Group 1 displayed notable changes with an increase in GM-CSF, TNFα, IFNγ, IL5, IL10, IL13, IL9, IL21, and IL22, and a significant decrease in IL4 and IL6. Group 2 did not show any prominent changes. Group 3 indicated a reduction in IFNγ, IL6, IL13, and IL17, coupled with an increase in IL9, IL21, and IL22.

Regarding the 3rd versus 2nd administration: Group 1 exhibited a decrease in GM-CSF, IFNγ, IL4, IL5, IL6, IL10, IL9, IL17, and IL21, with an increase in IL1β. Group 2 had a decline in GM-CSF, IL12, IL4, and IL10, and an elevation in IL5. Group 3 revealed an upward trend in IFNγ, IL9, IL17, IL21, and IL22, and a downward trend in IL12. 

Lastly, for the 3rd versus 1st administration comparison, Group 3 displayed a marked increase in IL2 and IFNγ, emphasizing the differences in cytokine profiles among the investigated groups.

## 3. Methodology

### 3.1. Patient Selection and Evaluation

Between January 2018 and January 2022, our study enrolled 80 patients diagnosed with autism, adhering to the Diagnostic and Statistical Manual of Mental Disorders, Fifth Edition (DSM-5) criteria for ASD [33]. Initial clinical evaluations were performed by a certified psychologist and a pediatric neurologist. For younger participants, aged between 24 and 36 months, the Ages and Stages Questionnaire-3 (ASQ-3) was employed. This developmental screening tool evaluates subjects across five domains: communication, gross motor skills, fine motor skills, problem-solving abilities, and personal–social interactions [34,35]. Based on the outcomes, patients were categorized into low-risk or high-risk ASD groups. It’s noteworthy that all subjects from this cohort were older than 36 months by the time of the six-month follow-up, making them eligible for the Gilliam Autism Rating Scale-3 (GARS-3) [36] assessment. For subjects aged 37 months to 17 years, the GARS-3 was utilized. This norm-referenced instrument assesses individuals with severe behavioral challenges that may be indicative of autism, focusing on six characteristic domains: stereotyped behaviors, communication, emotional responses, cognitive style, maladaptive speech, and social interactions. The GARS-3 classifies autism severity into three levels:

Level 0 (Autism Index ≤ 54): “Unlikely”

Level 1 (Autism Index 55–70): “Likely with minimal support”

Level 2 (Autism Index 71–100): “Very likely with substantial support”

Level 3 (Autism Index ≥ 101): “Very likely with intensive support”.

All participants in our study fell within levels 1–3, confirming their autism diagnosis. As part of our procedure, each child’s autism traits were further assessed using the GARS-3 by a special educator [35]. This tool has three primary subscales: (1) restrictive and repetitive behaviors, (2) social interaction, and (3) social communication. Notably, GARS-3 was employed both pre- and post-therapy, allowing us to monitor changes in patients’ scores.

Our rationale for employing GARS-3 as our primary classification tool encompasses:

Localized Translation: The GARS-3 was expertly translated into the subjects’ native language at the Medical College, University of Belgrade. This translation ensured a higher quality of responses, given the enhanced comprehension by patients and their parents.

Expert Validation: Medical professionals confirmed all test responses. Their expertise allowed the detection of nuanced changes and clarification of any ambiguities in the diagnostic or monitoring processes.

Longitudinal Assessment: We have a ten-year yearly repetition protocol for the GARS-3. Some of our subjects had already been under observation for multiple years prior to this study’s commencement.

#### 3.1.1. Assessing Child Development and Sensory Features

The Learning Accomplishment System Diagnostic (LAP-D) was employed to gauge each child’s developmental progress. Serving as a robust screener, the LAP-D determines potential risks associated with developmental delays. It offers a comprehensive assessment across various developmental stages, examining areas such as gross motor function, fine motor skills, cognitive abilities, expressive and receptive language, social behavior, and self-care skills [37]. Furthermore, to better understand the sensory processing patterns of children with ASD, we utilized the Short Sensory Profile (SSP) [38]. The SSP offers a standardized method for assessing how children with ASD process sensory information in diverse settings like homes, schools, and community activities.

#### 3.1.2. Evaluating Functional Outcomes Post-Transplantation

To assess the functional outcomes after autologous BMAC transplantation, we employed a patient grading system based on symptoms improvement. The gradations were categorized as:

Absent: No observable improvement.

Mild: Improvement in fewer than 30% of the symptoms.

Moderate: Improvement observed in 31%–80% of the symptoms.

Major: Enhancement in more than 81% of the symptoms.

Symptoms monitored encompassed stereotypical behaviors, communication, social interactions, tactile sensitivity, olfactory sensitivity or heightened reactions to stimuli, auditory filtering, visual/auditory sensitivity, energy levels, movement sensitivity, food consumption patterns, mobility, toilet independence, cognitive clarity, fine and gross motor skills, graphomotor abilities, and self-care.

#### 3.1.3. Exclusion Criteria for the Study

Potential participants were excluded from the study if they had epilepsy, hydrocephalus with a ventricular drain, coagulation disorders, allergies to anesthetic agents, or severe medical conditions. These severe conditions included active infections, cancer, and critical failures of the heart, blood, lungs, liver, or kidneys.

### 3.2. Autologous BMAC Derivation Process

Our approach to intrathecal autologous BMAC administration adhered to the Orto-MD-Parks protocol, segmented into three distinct phases:

1. Preliminary Testing: This phase is geared towards identifying active infections and any potential contraindications to anesthesia. Essential tests encompass comprehensive blood and urine analyses, chest radiographs, magnetic resonance imaging, and brain electroencephalography.
2. Bone Marrow Aspiration and Processing: Each participant received three intrathecal administrations of autologous BMAC. The initial injection followed immediately post-BM aspiration and processing. Subsequent injections were scheduled 30 days apart. The entire cell therapy process was completed within a day. Aspirations were performed under the purview of certified anesthesiologists using general anesthesia. The procedural specifics entailed positioning patients prone, making a precise incision on their right anterior iliac crest, and aspirating bone marrow through the iliac crest using a specialized 22G harvest needle. To anticoagulate the bone marrow, we utilized the Acid Citrate Dextrose (ACD) formula A in a 7:1 ratio [31]. The Angel whole blood separation system (Arthrex, Naples, FL, USA) was instrumental in processing bone marrow. Through density gradient centrifugation, we successfully separated the BMAC, hematopoietic stem cells, and platelet-poor plasma. The extracted volume was tailored to the patient’s weight, typically 8 mL/kg. For those weighing below 10 kg, it was restricted to 4–5 mL/kg, with an overall cap of 160 mL. Subsequently, we quantified the BMAC, hematopoietic stem cells (CD34+), and platelet-poor plasma.
3. BMAC Administration: We favored intrathecal (IT) autologous BMAC administration due to its less invasive nature in contrast to direct brain delivery, while still ensuring effective brain delivery. Throughout our study’s monitoring phase, we observed no severe adverse reactions post intrathecal autologous BMAC administration. Any mild adverse events typically subsided within a two-hour window, affirming the procedure’s safety.

### 3.3. Autologous BMAC Isolation

Following the extraction process, cell quantities were assessed using the Beckman Coulter AcT diff cell counter, standardizing the count to 5 × 10^6^ cells/mL of the suspension. A blend of monoclonal antibodies, targeting Stro-1, CD133, CD73, CD146, CD105, CD45, CD34, CD90, and 7AAD, was employed for cell staining. Cytometric analysis was then utilized to ascertain the viability and quantity of both BMAC and hematopoietic stem cells (CD34+).

### 3.4. Cytokine Levels and Cell Viability Assessment

We selected cytokines known to influence the inflammatory processes and enhance the MSCs’ immunomodulatory influence, Table 8. For cytokine analysis, we utilized the volume of CSF aspirated, which equaled the administered BMAC volume prior to each application. Cytokines assessed in both BMAC and CSF encompassed Th1 (IL2, IL12, IFNγ), Th2 (IL4, IL5, IL6, IL10, IL13, IL9) TNFα, IL17, IL21, IL22, IL27, GM-CSF, and IL1β. Additionally, levels of the markers Stro-1, CD133, CD73, CD146, CD105, CD45, CD34, CD90, and 7AAD were determined.

Flow cytometry was employed to verify the viability and quantify BMAC and hematopoietic stem cells (CD34+). During administration, these cells were introduced intrathecally into the subarachnoid space. For the three intrathecal injections, the average total nucleated cell (TNC) counts and viability percentages were as follows:

1st injection: TNC of 56 × 10^6^ mL and 98% viability.

2nd injection: TNC of 49 × 10^6^ mL and 99% viability.

3rd injection: TNC of 52 × 10^6^ mL and 99.6% viability.

### 3.5. BMAC Transplantation Procedure

The initial BM count of the BMAC was established as per standard guidelines [39]. Prior to the intrathecal administration of BMAC, we meticulously prepared the injection site and extracted an equivalent volume of CSF. This step ensures the maintenance of CSF’s natural circulation during transplantation. The BMAC suspension was then intrathecally injected between the fourth and fifth lumbar vertebrae using a 20G spinal needle, spanning a procedure time of approximately 30 min.

To quantify the absolute number of specific cells in the BMAC sample, we combined the percentage of CD90-positive cells with the total cell count in the sample. These data provided a reference benchmark, aiding in discerning any increase in subsequent measurements. Statistical analysis was conducted using Friedman’s test for paired samples.

For ethical considerations, **the baseline CSF cytokine levels—gathered prior to the initial BMAC transplantation—acted as the control**, eliminating the need for a separate control group. Consequently, each participant also functioned as their own control. Follow-up CSF analyses were scheduled a month post the first administration, and again two months after the inaugural BMAC transplantation.

### 3.6. Evaluation after BMAC Therapy

We analyzed cytokine levels in the CSF samples during every BMAC administration. To ensure the safety of stem cell transplantation and the multiple BMAC treatments, we vigilantly monitored for any long-term adverse events. Notably, there were no instances of significant adverse events or seizures among the patients. The side effects that did occur were minor and short-lived, including symptoms like nausea, pain at the injection or aspiration sites, and vomiting. These were resolved within two hours and were primarily linked to the intrathecal procedure rather than the transplantation of bone marrow cells.

Post-therapy, patients underwent comprehensive neurorehabilitation. This included occupational therapy, psychological therapy, behavior analysis, sensory integration, and speech therapy. After each BMAC administration, certified psychologists and pediatric neurologists conducted rigorous follow-ups using the LAP-D scoring system.

To assess the therapeutic outcomes, we implemented a structured grading system. This system categorized the observed improvements in symptoms as absent/mild, moderate, or major. Of the 80 initial participants, 59 returned for a comprehensive six-month follow-up with a pediatrician. Using the ASD scoring frameworks, these patients were segregated into three distinct groups based on their therapeutic response. Group 1 comprised 28 patients showing minimal to no improvements post autologous BMAC therapy, Group 2 included 16 patients who displayed moderate improvements, while Group 3 had 15 patients who exhibited significant therapeutic benefits.

### 3.7. Statistical Analysis

The Shapiro–Wilk test was used to test the normality of the variables. The Wilcoxon test was used to compare cytokine concentration differences between two clinical response groups with values expressed as a mean concentration and a standard deviation and between the two-time intervals for a single group. The changes in cytokine concentration between the follow-ups and between the groups were estimated using the Kruskal–Wallis, Dunn’s multiple comparison post-tests. Data analyses were performed using commercial software (Prism 5.0, GraphPad Software, Inc., La Jolla, CA, USA). Encompassing multi-facets of ASD, we adopted a synergistic approach to statistical analysis to capture a robust overview of cytokine differences across the Groups, and inherent temporal dynamics associated with multiple stem cell administrations. Thus, we were able to account for both immediate and cumulative effects of the treatment.

## 4. Discussion

The primary objective of our study was to analyze the association between early improvements after autologous BMAC transplantation and the cytokine profile. Our results show a significant association between cytokine levels and decreased severity of autism symptoms. Significant symptom improvements were observed in body posture, intellectual response, visual response scores, taste, smell, touch scores, and fear or nervous scores. To the best of our knowledge, this study has the largest group of male and female children investigating the relationship between clinical traits of ASD with autologous BMAC therapy and cytokine levels after three intrathecal administrations. The study examined what cytokine types had altered CSF concentration levels and which types were linked with ASD severity and improvement before, during, and after BMAC therapy. Note that we did not study the influence of cytokine levels on an individual symptom but on the general clinical picture of ASD. Our results revealed several associations between the concentrations of cytokines and clinical improvements.

Our study confirmed that cytokine production was associated with altered behavior in autistic children: increased proinflammatory or Th1 cytokines were associated with decreased clinical improvements. The production of GM-CSF and Th2 cytokines was associated with better cognitive and adaptive function. The results indicate that the CSF baseline average level of IL27 is significantly lower in the children whose autologous BMAC treatment did not improve the autism score (Group 1). IL27 may modulate autoimmune inflammation via promotion of the Treg lineage, and it often competes against the action of IL-632. In our study, Group 1 demonstrated a significantly lower IL27 concentration in the baseline, and Group 3 showed a high IL27 baseline concentration. Several studies have verified significantly higher serum IL17 in ASD patients and confirmed an association between cytokine serum levels and disease severity [40]. IL17 might contribute to autism symptoms by inhibiting neural stem cell differentiation, inducing microglia, altering the blood–brain barrier (BBB) permeability, inducing apoptosis in oligodendrocytes, and increasing glutamate levels and excitotoxicity [41]. Our study demonstrated similar IL17 baseline levels in all three groups. However, after the first BMAC procedure, a significant increase was noted in Group 1. Group 3 had the opposite results, i.e., a decreased average CSF concentration of IL17 cytokine after the first BMAC administration. TNF is a multifunctional cytokine required for brain cell maintenance and homeostasis, and it is constitutively secreted in minute amounts by neurons and glia. Xie et al. [42] positively correlated TNF-α concentration with five subscales of the autism symptoms score, providing evidence that TNF-α blood concentrations may act as ASD biomarkers. TNF- α can cross from the peripheral blood into the brain and thus directly affect brain function via their receptors [43].

Our findings confirmed that IL1β is increased in the CSF and the brain of ASD patients [43,44,45,46]. Increased levels of IFN-γ can induce inflammation. Eftekhariana et al. [47] and Pardo et al. [48] investigated the IFN-γ serum concentration of ASD patients and confirmed no difference compared to healthy controls. However, their data could not be discussed in comparison to our finding that Group 3 demonstrated the decreased average concentration of IFN-γ and IL1β in CSF since our study reflects local immune responses after the treatment.

Our results connote essential links between CSF cytokine levels and early improvements in ASD symptoms: regulation of cytokine patterns contributes to significant improvements in the child’s behavior during the first six months. This implies the successful homing [49] of autologous BMAC and cytokines to the brain, where they acted on targeted cell types. In the brain, this immunological influx triggered neuroplasticity [50].

A good understanding of the primary mechanisms is essential to realizing MSCs’ therapeutic potential. Our study implies that the three BMAC administrations contribute to cytokine-levels regulation. Despite some encouraging preliminary progress, the study opens several important technical points, such as 1. What is the long-lasting effect of the treatment? 2. What is the maintenance timeline? 3. More detailed definition of subgroups, focusing on ASD subgroup in terms of neurocognitive profiles, IQ, adaptive skills, and other detailed behavioral profiles, i.e., replication of the work in a refined and larger ASD sample; 4. IT-delivered BMAC is well tolerated with no major issues, and thus deserves further study, focusing on the optimal stem cell doses and the frequency of administration; 5. Lacking normative data on cytokines in the general population and highly heterogeneous ASD subpopulations suggests the need for additional studies to fully understand and interpret the baseline and cytokine changes reported in this paper; 6. Higher resolution timeline is needed to determine the exact timing of post-treatment cytokine changes.

The regulation of cytokine levels is a potential future therapeutic target. The presented results showcase the crucial role of cytokines in autism. However, we still need to learn how to deploy cytokines for the early identification of autism. It remains unclear which specific cytokines have the highest priority for improving autism symptoms and whether different concentrations of cytokines are associated with corresponding changes in autistic behavior. Further studies are necessary to determine a link between improvements in autism symptoms and elevated/depressed cytokine levels. Future work will focus on the role of cytokines as markers sensitive to a response to autism cell therapies. The improvements in monitored symptoms point to triggered brain neuroplasticity. However, what is not clear is whether this is triggered functional neuroplasticity, i.e., the brain’s ability to dislocate the functions from the dysfunctional area to other functional areas, or structural plasticity, i.e., actual changes in the brain’s physical structure. The confirmed connection between CSF cytokine levels and early improvements in autism symptoms suggests that cytokine profiles before treatment could be a predictive indicator of treatment success and a guiding marker for treatment decisions and monitoring.

### Clinical Implications of Cytokine Dynamics in BMAC Therapy for ASD

Previous studies have confirmed the positive healing promise and safety of cell therapies in ASD patients (Table 9) [27,31,45,46,47,48,49,50]. However, it is hard to qualitatively compare the results between the studies due to the deployment of different evaluation metrics, cell therapy protocols, and cultural and post-treatments rehabilitation differences that may influence the results between the follow-up periods. For example, our findings, the case report described in [27], and the results in 31] point to the importance of physical rehabilitation as post-treatment therapy. Given the link between ASD symptoms and cytokine profiles, understanding how these therapies influence cytokine dynamics could be pivotal. Note that although Sharifzadeh et al. [51] reported no significant differences between the control and intervention groups in the Childhood Autism Rating Scale (CARS) total score, GARS-II autism index, or Clinical Global Impression (CGI) global improvement over 12 months, they also recorded that the CGI severity of illness score showed significantly greater improvement in the marrow-derived mononuclear cells (BMMSC) group, and two CARS subscales also showed significantly greater improvement in the BMMSC group. Incorporating cytokine analysis in these studies might provide a clearer picture of the underlying mechanisms at play. For instance, if BMAC therapy alters cytokine profiles in a way that reduces proinflammatory cytokines, this could explain the observed improvements in certain clinical metrics. The cytokine analyses would have given deeper insights into these scores. Note that BMAC is a more concentrated subset of BMMSC.

This study concurs and provides compelling evidence that autologous BMAC therapy may improve ASD behavioral symptoms through immunomodulation and altering cytokine signaling patterns in the CNS. It aligns with the emerging understanding that the neuroinflammatory environment, particularly cytokine activity, plays a fundamental role in the manifestation and severity of ASD symptoms. Patients exhibiting the greatest clinical gains displayed decreased proinflammatory and Th1 cytokine levels in CSF following BMAC treatment.

While prior studies have associated ASD with immune system disturbances, our findings of cytokine level correlations with behavioral responses following an immunomodulatory intervention are novel. This suggests cytokine profile changes are not merely an epiphenomenon but may play a mechanistic role in BMAC efficacy. Modulating cytokine networks appears to be a primary pathway by which transplanted BMAC impacts the brain.

Our results indicate that CSF cytokine levels may have clinical utility as predictive biomarkers for identifying ASD patients most likely to benefit from BMAC therapy on an individualized basis. Patients with no/mild gains had lower baseline IL27, while major responders displayed elevated IL27. IL27 regulates inflammatory T-cell responses, so may relate to treatment-induced anti-inflammatory effects.

Demographically, this study is in line with the observation that ASD prevails in males. The study also provides reassurance that intrathecal delivery of autologous BMAC is well-tolerated, with no major adverse events and only transient side effects. This safety profile supports further research into optimized BMAC dosing regimens and long-term monitoring. While the BMAC study offers promising insights into a potential therapeutic avenue for ASD, understanding its impact within the framework of cytokine dynamics could be the key to unlocking its full potential. Our research on cytokine dynamics in autism highlighted the intricate relationship between specific cytokine profiles and the neuroinflammatory responses inherent in ASD. Assessing cytokine profiles before and after BMAC treatment could provide valuable insights into the treatment’s mechanism of action and help refine patient selection for maximum therapeutic benefit.

Future directions should focus on validating our findings in larger randomized controlled trials across heterogeneous ASD populations. Subgrouping patients based on cognitive profiles and behavioral phenotypes may reveal further biological insights. Larger samples can help establish normative cytokine data to aid interpretation.

## Figures and Tables

**Table 1:** Demographics and GARS-3 score distribution of the subjects.

Demographic Characteristics and Score	Demographic Group and Levels	No. of Patients (N = 80)
Gender	male	63
	female	17
Age	24–36 months	6
	37 months–16 years	72
	17 years	2
GARS-3 score	Level 1	10
	Level 2	68
	Level 3	2

**Table 2:** Average concentrations and standard deviations (SD) of cytokines examined in CSF samples from children with autism, presented as x ± SDx ± SD in pg/mL. The procedure’s number indicates the round of autologous BMAC administration, while “GROUP” refers to the classification of patient improvement six months post-therapy, as detailed in the main text, Section 2.1. Please note that we have denoted significant statistical differences between groups per procedure using lowercase letters, to be consistent with additional details in Table 3. For example, the average TNFα concentrations in Group 1 and Group 3 during the 2nd administration are labelled with the letter “a”, implying significant statistical differences in TNFα concentrations between Groups 1 and 3.

	1st Procedure			2nd Procedure			3rd Procedure		
Group	1	2	3	1	2	3	1	2	3
GM-CSF	293 ± 557	383 ± 729	511 ± 1082	344 ± 629	408 ± 675	108 ± 192	199 ± 416	193 ± 416	418 ± 1069
TNFα	35 ± 26	36 ± 31	36 ± 26	**49 ± 24** **^a^**	**54 ± 37** **^b^**	**27 ± 18** **^a,b^**	41 ± 23	39 ± 34	33 ± 31
IL1β	23 ± 26	23 ± 19	17 ± 17	**37 ± 28** **^c^**	35 ± 34	**17 ± 21** **^c^**	28 ± 22	24 ± 19	17 ± 21
IL2	44 ± 42	54 ± 46	44 ± 29	**71 ± 41** **^d^**	**92 ± 63** **^e^**	**41 ± 36** **^d,e^**	53 ± 37	49 ± 33	47 ± 48
IFNγ	54 ± 66	62 ± 61	51 ± 44	**88 ± 69** **^f^**	**98 ± 93** **^g^**	**38 ± 31** **^f,g^**	76 ± 70	71 ± 57	66 ± 83
IL12	26 ± 26	24 ± 20	24 ± 20	**34 ± 19** **^h^**	34 ± 26	**20 ± 18** **^h^**	29 ± 21	36 ± 23	27 ± 24
IL4	522 ± 561	515 ± 672	422 ± 543	514 ± 568	512 ± 648	275 ± 279	386 ± 532	259 ± 512	389 ± 678
IL5	54 ± 85	63 ± 77	74 ± 120	83 ± 78	115 ± 141	48 ± 65	81 ± 84	118 ± 127	91 ± 141
IL6	103 ± 143	143 ± 145	101 ± 114	158 ± 168	228 ± 264	95 ± 104	128 ± 120	191 ± 176	122 ± 144
IL10	28 ± 30	28 ± 26	34 ± 28	37 ± 26	43 ± 32	25 ± 25	36 ± 30	37 ± 19	35 ± 33
IL13	81 ± 119	78 ± 86	72 ± 69	**145 ± 135** **^i^**	123 ± 127	**59 ± 72** **^i^**	99 ± 117	107 ± 106	89 ± 141
IL17	88 ± 107	86 ± 79	84 ± 81	**143 ± 103** **^j^**	170 ± 188	**71 ± 80** **^j^**	99 ± 90	96 ± 66	103 ± 132
IL9	14 ± 20	23 ± 21	14 ± 18	26 ± 27	34 ± 34	16 ± 20	19 ± 19	27 ± 24	22 ± 28
IL21	15 ± 25	19 ± 23	10 ± 21	29 ± 35	**40 ± 40** **^k^**	**15 ± 23** **^k^**	22 ± 26	29 ± 27	25 ± 35
IL22	4 ± 6	7 ± 7	3 ± 6	7 ± 7	9 ± 8	4 ± 6	7 ± 7	7 ± 8	7 ± 8
IL27	**10 ± 10** **^l,m^**	**15 ± 6** **^l^**	**15 ± 5** **^m^**	14 ± 8	16 ± 11	14 ± 7	13 ± 6	12 ± 8	11 ± 8

**Table 3:** Statistical analysis using the Wilcoxon test of average cytokine concentrations between the Groups per procedure, with small letters indicating significant differences from Table 1. If a *p*-value is below 0.05, it is marked with one star (*), and if it is below 0.01, it is marked with two stars (**).

Cytokine	*p*	Sig	Procedure	Group	vs	Group
TNFα	*p* = 0.0082	** ^a^	2nd	1	>	3
IL1β	*p* = 0.0443	* ^c^	2nd	1	>	3
IL2	*p* = 0.0400	* ^d^	2nd	1	>	3
IFNγ	*p* = 0.0238	* ^f^	2nd	1	>	3
IL12	*p* = 0.0373	* ^h^	2nd	1	>	3
IL13	*p* = 0.0440	* ^i^	2nd	1	>	3
IL17	*p* = 0.0456	* ^j^	2nd	1	>	3
TNFα	*p* = 0.0405	* ^b^	2nd	2	>	3
IL2	*p* = 0.0306	* ^e^	2nd	2	>	3
IFNγ	*p* = 0.0460	* ^g^	2nd	2	>	3
IL21	*p* = 0.0483	* ^k^	2nd	2	>	3
IL27	*p* = 0.0209	* ^l^	1st	2	>	1
IL27	*p* = 0.0494	* ^m^	1st	3	>	1

**Table 4:** Statistical analysis of serial samples per Group between the procedure rounds using the Wilcoxon test. The red triangle denotes a significant increase in the cytokine. If a *p*-value is below 0.05, it is marked with one star (*).

Cytokine	Group	2nd/1st	3rd/2nd	3rd/1st
GM CSF	1	>0.05	>0.05	>0.05
	2	>0.05	>0.05	>0.05
	3	>0.05	>0.05	>0.05
TNFα	1	>0.05	>0.05	>0.05
	2	>0.05	>0.05	>0.05
	3	>0.05	>0.05	>0.05
IL1β	1	>0.05	>0.05	>0.05
	2	>0.05	>0.05	>0.05
	3	>0.05	>0.05	>0.05
IL2	1	▲ *p* = 0.0345 *	>0.05	>0.05
	2	>0.05	>0.05	>0.05
	3	>0.05	>0.05	>0.05
IFNγ	1	▲ *p* = 0.0170 *	>0.05	>0.05
	2	>0.05	>0.05	>0.05
	3	>0.05	>0.05	>0.05
IL12	1	▲ *p* = 0.0263 *	>0.05	>0.05
	2	>0.05	>0.05	>0.05
	3	>0.05	>0.05	>0.05
IL4	1	>0.05	>0.05	>0.05
	2	>0.05	>0.05	>0.05
	3	>0.05	>0.05	>0.05
IL5	1	>0.05	>0.05	>0.05
	2	>0.05	>0.05	>0.05
	3	>0.05	>0.05	>0.05
IL6	1	>0.05	>0.05	>0.05
	2	>0.05	>0.05	>0.05
	3	>0.05	>0.05	>0.05
IL10	1	▲ *p* = 0.0485 *	>0.05	>0.05
	2	>0.05	>0.05	>0.05
	3	>0.05	>0.05	>0.05
IL13	1	▲ *p* = 0.0459 *	>0.05	>0.05
	2	>0.05	>0.05	>0.05
	3	>0.05	>0.05	>0.05
IL9	1	▲ *p* = 0.0485 *	>0.05	>0.05
	2	>0.05	>0.05	>0.05
	3	>0.05	>0.05	>0.05
IL17	1	▲ *p* = 0.0495 *	>0.05	>0.05
	2	>0.05	>0.05	>0.05
	3	>0.05	>0.05	>0.05
IL21	1	>0.05	>0.05	>0.05
	2	>0.05	>0.05	>0.05
	3	>0.05	>0.05	>0.05
IL22	1	>0.05	>0.05	>0.05
	2	>0.05	>0.05	>0.05
	3	>0.05	>0.05	>0.05
IL27	1	>0.05	>0.05	>0.05
	2	>0.05	>0.05	>0.05
	3	>0.05	>0.05	>0.05

**Table 5:** Comparisons between groups per administration (Kruskal–Wallis test, Dunn’s Multiple Comparison post-test). If a *p*-value is less than 0.05, it is flagged with one star (*). If a *p*-value is less than 0.01, it is flagged with 2 stars (**).

	1st Administration			2nd Administration			3rd Administration		
Cytokine	Gr1/Gr2	Gr1/Gr3	Gr2/Gr3	Gr1/Gr2	Gr1/Gr3	Gr2/Gr3	Gr1/Gr2	Gr1/Gr3	Gr2/Gr3
GM-CSF					**				
TNFα					*				
IL1β					**				
IL2					**	*			
IFNγ					**				
IL12					*				
IL4									
IL5									
IL6									
IL10					*	*			
IL13					*				
IL17					*				
IL9									
IL21					*				
IL22									
IL27									

**Table 6:** Comparisons between procedures in each patient group (Kruskal–Wallis test, Dunn’s Multiple Comparison post-test). If a *p*-value is less than 0.05, it is flagged with one star (*). If a *p*-value is less than 0.01, it is flagged with 2 stars (**).

	Group 1.			Group 2.			Group 3.		
Cytokine	1st/2nd	1st/3rd	2nd/3rd	1st/2nd	1st/3rd	2nd/3rd	1st/2nd	1st/3rd	2nd/3rd
GM-CSF	*								
TNFα									
IL1β									
IL2	**								
IFNγ	**								
IL12									
IL4									
IL5									
IL6									
IL10									
IL13									
IL17	*								
IL9									
IL21									
IL22									
IL27	*								

**Table 7:** Cytokine Dynamics: direction of changes across administration intervals: decrease (blue triangle), increase (red triangle), or no change (=). *p*-values are rated using a star system, with one star (*) indicating *p* < 0.05, two stars (**) for *p* < 0.01, three stars (***) for *p* < 0.001, four stars (****) for *p* < 0.0001, and “ns” representing “Not Significant”.

2nd/1st Administration	Group 1	Group 2	Group 3
GM CSF	▲ ***	ns	ns
TNFα	▲ **	ns	ns
IL1β	ns	ns	**=** **
IL2	ns	ns	ns
IFNγ	▲ *	ns	▼ ***
IL12	ns	ns	ns
IL4	▼ ***	ns	ns
IL5	▲ **	ns	ns
IL6	▼ *	ns	▼ **
IL10	▲ **	ns	ns
IL13	▲ **	ns	▼ *
IL9	▲ *	ns	▲ **
IL17	ns	ns	▼ *
IL21	▲ *	ns	▲ *
IL22	▲ *	ns	▲ **
IL27	ns	ns	ns
**3rd/2nd** **administration**	**Group 1**	**Group 2**	**Group 3**
GM CSF	▼ ****	▼ ****	ns
TNFα	ns	ns	ns
IL1β	▲ **	ns	**=** *
IL2	ns	ns	ns
IFNγ	▼ *	ns	▲ *
IL12	ns	▼ *	▼ *
IL4	▼ ***	▼ ***	ns
IL5	▼ **	▲ ***	ns
IL6	▼ **	ns	ns
IL10	▼ *	▼ *	ns
IL13	ns	ns	ns
IL9	▼ **	ns	▲ *
IL17	▼ *	ns	▲ *
IL21	▼ *	ns	▲ *
IL22	= *	ns	▲ **
IL27	ns	ns	ns
**3rd/1stadministration**	**Group 1**	**Group 2**	**Group 3**
GM CSF	▼ ****	ns	ns
TNFα	ns	ns	ns
IL1β	ns	ns	**=** *
IL2	ns	ns	▲ *
IFNγ	ns	ns	▲ *
IL12	ns	ns	ns
IL4	▼ ***	ns	ns
IL5	▲ **	ns	ns
IL6	ns	ns	▲ **
IL10	▲ *	ns	ns
IL13	ns	▼ *	ns
IL9	ns	ns	▲ *
IL17	▲ **	ns	▲ **
IL21	▲ *	ns	ns
IL22	▲ *	ns	▲ **
IL27	ns	ns	ns

**Table 8:** Cytokines: Inflammatory Classification and Roles. The table reflects the dual roles of some cytokines in terms of their inflammatory classification and the various functions they can perform in different contexts. Note that the classification of some cytokines may depend on specific contexts and interactions within the immune system, and their functions can sometimes be more complex than a simple pro or anti-inflammatory label.

Cytokine	Inflammatory Classification	Other Roles
GM-CSF	Pro-inflammatory	Hematopoietic growth, Immune regulation
TNFα	Pro/anti-inflammatory	Immune regulation, Neuroprotection
IL1β	Pro-inflammatory	Fever induction, Tissue repair
IL2	Pro-inflammatory	T-cell growth, Immune tolerance
IFNγ	Pro-inflammatory	Antiviral response, Tissue repair
IL12	Pro-inflammatory	T-cell differentiation, Immune regulation
IL4	Anti/Pro-inflammatory	B-cell activation
IL5	Anti/Pro-inflammatory	Eosinophil activation
IL6	Pro/anti-inflammatory	Immune regulation
IL10	Anti/Pro-inflammatory	Immune regulation
IL13	Anti/Pro-inflammatory	Tissue repair, Pro-inflammatory
IL17	Pro-inflammatory	Neutrophil recruitment, Immune regulation
IL9	Pro-inflammatory	Allergic responses, Immune regulation
IL21	Pro-inflammatory	B-cell differentiation, Immune regulation
IL22	Pro-inflammatory	Epithelial cell repair, Immune regulation
IL27	Anti/Pro-inflammatory	Immune regulation

**Table 9:** Clinical effectiveness of stem cell transplantation in ASD patients.

Authors	Type	Administration Route	# of Patients	Improvement	Safety
Sharma et al., 2013 [52]	Autologous BM-MNC GM CSF ind, CD34 isolated	Intratechal	32p	29 (91%)	YES
Lv et al., 2013 [53]	Cord blood MNCUmb blood-derived MSC	IntravenousIntratechal	23p + 14c	No Diff p/c20–60% before/after score	YES
Bradstreet et al., 2014 [54]	Fetal SC	IntravenousSubcutaneous	45p	35 (78%)	YES
Chez et al., 2018 [55]	Autologous umbilicalCord blood	Intravenous	29p	5–24% bef/aft score	YES
Sharifzadeh et al., 2020 [51]	Autologous BM-MNC	Intratechal	14p + 18c	No Diff p/c	YES
Sharma et al., 2020 [56]	Autologous BM-MNC	Intratechal	254p	50–82%	YES
Sharma et al., 2023 [57]	Autologous BM-MNC	Intratechal	1011p	53–87%	YES

## Data Availability

The datasets collected or analyzed during the current study are available from the corresponding author upon reasonable request.
